# Assessing the effect of introducing a new method into family planning programs in India, Peru, and Rwanda

**DOI:** 10.1186/1742-4755-9-17

**Published:** 2012-09-01

**Authors:** Rebecka Lundgren, Irit Sinai, Priya Jha, Marie Mukabatsinda, Luisa Sacieta, Federico R León

**Affiliations:** 1 Institute for Reproductive Health, Georgetown University, 4301 Connecticut Ave, NW, Washington DC, USA; 2 Institute for Reproductive Health, New Delhi, India; 3 Institute for Reproductive Health, Kigali, Rwanda; 4 Instituto para la Salud Reproductiva, San Miguel, Peru; 5 León & Bustamante Consultores, Lima, Peru

**Keywords:** Family planning, Standard Days Method, Fertility awareness, New method introduction

## Abstract

**Background:**

Introducing a new method into family planning programs requires careful attention to ensure it meets an actual need and has a positive effect on program goals. The Standard Days Method® is a fertility awareness-based method of family planning that is being introduced into family planning programs in countries around the world. It is different from other methods offered by programs, and may bring new couples into family planning, and increase contraceptive prevalence. The study assesses the effect on contraceptive use and prevalence of Introducing Standard Days Method into existing family planning services in whole regions of India, Peru, and Rwanda.

**Methods:**

In collaboration with the Ministry of Health, health providers were given a contraceptive update on all methods, then trained in counseling on Standard Days Method. Efforts were made to promote demand in the context of informed choice. Routine monthly service statistics in control and intervention areas were used to assess the effect of Standard Days Method introduction at the clinic level; baseline and endline household-based surveys were undertaken to obtain results at the community level (n > 3400 women at endline).

**Results:**

Demand for the method is evident in countries with different levels of contraceptive prevalence. The method attracts couples new to family planning, and introducing it into services may increase overall contraceptive prevalence.

**Conclusions:**

Introducing Standard Days Method into existing family planning has the potential of benefiting men and women in diverse settings and populations. This study illustrates the critical role of evidence in scaling up a health innovation.

## Background

Contraceptive choice and access to family planning are key to achieving the Millennium Development Goals of reduced child mortality and improved maternal health, and can contribute to reduced poverty [1]. Evidence suggests that when a new contraceptive method is added to the mix, it attracts new clientele and increases contraceptive prevalence [2]. Jain (1989) has estimated that the widespread addition of one method to options available in a country would be associated with an increase of 12% in overall contraceptive prevalence [3]. Understanding the programmatic context is critical [4], and concerns of key stakeholders must be addressed [5]. Scaling-up the introduction of a new method presents a particular challenge, as the larger environment cannot be controlled to the same extent as during pilot introduction [6]. Attention must be given to how the new method responds to peoples’ needs and rights. The innovation must be adapted to the local context in a participatory approach that includes local stakeholders and policy makers, systematic use of evidence, and an ongoing focus on sustainability [7].

This study examines the introduction of Standard Days Method® into existing services in large regions in India, Peru, and Rwanda, that involved adding the method into services offered by the Ministry of Health (MOH) and some non-governmental organizations (NGOs) in selected regions. We report results from service statistics and community surveys designed to evaluate this effort and respond to several key questions asked by stakeholders. Specifically, the study was designed to assess:

What proportion of women and men in the community are aware of Standard Days Method? This measures the success of communication and outreach strategies, an important consideration in scaling-up the method;

How many clients accept Standard Days Method, compared to established methods? This measures the method’s ability to respond to clients’ perceived needs;

Does introducing Standard Days Method increase overall family planning use in MOH facilities and in the community? This measures the effect of the method on contraceptive prevalence;

Are Standard Days Method users new to family planning, or do they shift from established methods? This measures the method’s potential to reduce unmet need, and addresses a common stakeholder concern that new Standard Days Method users switch from other established methods.

### The Standard Days Method

Standard Days Method is a fertility awareness-based method of family planning that identifies days 8 to 19 of the menstrual cycle (inclusive) as the days when unprotected intercourse is more likely to result in pregnancy. To prevent pregnancy, users avoid unprotected intercourse during the 12-day fertile window. The method works best for women with cycles that usually range 26–32 days [8]. It is often offered with CycleBeads®, a set of color-coded beads that help users track their cycles and identify their fertile days.

Efficacy rates of Standard Days Method are comparable to those of male condoms and better than those of other barrier methods, with a pregnancy rate of 4.8 (per 100 women years) with correct use, and 12.0 with typical use [9]. A series of 14 strategically designed pilot studies in diverse settings around the world found demand for the method by a broad range of women. Users learned the method in a single visit, usually less than 30 minutes, and were generally satisfied with it. Most women (90%-99%) found the method easy to learn, simple to use, effective, and without side effects [10]. The method presents a viable longer-term option for women who prefer this approach to family planning [11]. The current study examines the effect on the community of larger-scale Standard Days Method introduction.

## Methods

The study intervention was replicated in three very different settings – India, Peru, and Rwanda – where the MOH was interested in introducing Standard Days Method into services. Specific regions in each country were selected in collaboration with national and local governments, and represent areas where donors and governments were interested in expanding access to Standard Days Method.

### Study sites

In India, the state of Jharkhand (population 27 million) was selected. Only 31% of married women of reproductive age in Jharkhand use any modern family planning (most commonly sterilization) [12]. Three blocks in Ranchi district of Jharkhand were included. Kanke and Ormanjhi were selected as the intervention sites; Burmu as control. Two Standard Days Method pilot studies were previously undertaken in India, but not in the current study areas.

Among the three countries included in this study, Peru has the highest contraceptive prevalence rate. Of the 70% of contracepting married women of reproductive age, 48% use a modern method; the rest use traditional methods, mainly a form of periodic abstinence [13]. Peru was one of three countries where Standard Days Method efficacy was tested [8], but not in the current study areas. Three adjacent health districts in Moyobamba province in San Martin, were selected for the intervention site; three adjacent districts in Jaén province (Cajamarca region) were the control.

While contraceptive prevalence has been fairly stable in Peru and India, the government of Rwanda is actively seeking to increase contraceptive prevalence, and aims to decrease the total fertility rate from 6.5 in 2000, to 4.5 by 2020 [14]. Modern contraceptive use almost doubled from 2000 to 2005, from 5.7% of married women to 10.3% (Rwanda National Population Office, and ORC Macro, 2001; 2006) [15,16]. The most widely used modern method in Rwanda is the contraceptive injection. As in India and Peru, Standard Days Method was offered in Rwanda before the current study; the method was introduced into regular service delivery (not part of a study) in partnership with the Rwanda MOH and the IntraHealth PRIME program in 13 pilot facilities in 2002. Two years later 15 additional facilities were added in seven of the country’s 12 provinces. The province of Byumba, selected for the intervention in this study, includes two of the 13 pilot facilities [17]. Kibungo province was selected as control, because it did not include any of the pilot facilities.

Table 1 summarizes the charteristics of the study sites and how they differ.

**Table 1:** Study sites

	**India, Jharkhand**		**Peru**		**Rwanda**	
	**Intervention**	**Control**	**Intervention**	**Control**	**Intervention**	**Control**
District or province	Kanke, Ranchi	Burmu, Ranchi	Moyobamba.San Martin	Jaén, Cajamarca	Byumba	Kibungu
	Ormanjhi, Ranchi					
# of service delivery points	66	23	35	32	20	19
# of trained providers	186 facility-based;	0	100 facility-based	0	133 facility-based;	0
	390 community-based				830 community-based*	
Presence of SDM prior	No	No	No	No	2 facilities	No
Baseline** survey date	December, 2004		January – March, 2005			
Endline survey date	November 2006 – January 2007		November 2006 – February 2007		October 2006	
	October – November, 2007					

* Community-based health workers in Rwanda could refer clients to health facilities, but not counsel clients directly.

** Intervention activities began everywhere immediately after baseline survey data collection.

### The intervention

The purpose of the intervention in all three countries was to make Standard Days Method accessible to couples in the intervention areas as an additional family planning option. Standard Days Method introduction included advocacy with key stakeholders such as MOH officials and medical and nursing associations that resulted in the inclusion of CycleBeads in the health management information and procurement and logistics systems; training providers (all providers of all levels who offer family planning in participating facilities) and supervisors in Standard Days Method counseling after updating them on all methods, monitoring and supervision, and information, education, and communication (IEC) activities.

Intervention activities varied somewhat based on each country’s social and political environment. A participatory approach, involving stakeholders, policy makers, and program managers, was used to determine the best strategy to include the method in training, supervision, and IEC efforts in each country. Introduction activities were monitored closely throughout [18], and adjusted as needed. An important mid-way change in India was adding family planning community workers as Standard Days Method providers a year after clinic-based providers began offering the method.

A two-day skills-based training was conducted in each country to enable providers to help clients determine whether Standard Days Method would work well for them, teach clients to use the method, explore approaches for effectively managing the fertile days, and encourage partner involvement. In the training participants were provided a handbook and job aids appropriate to their literacy level as well as a starter set of CycleBeads.

Standard Days Method was incorporated into supervision visits to reinforce knowledge, monitor recording Standard Days Method users in service statistics and ensure availability of CycleBeads. When possible, supervisors conducted role plays with providers using a simple structured check list to reinforce essential elements of method provision. In addition, providers discussed challenges and solutions related to Standard Days Method services during regular meetings, for example when community health workers visited the facility to pick up supplies or during quarterly program review sessions.

A one-day refresher training was conducted several months after providers had begun to offer Standard Days Method to address specific issues noticed during supervision, most notably the tendency of providers to unnecessarily require women to track their menstrual cycles before offering the method or to require male partners to participate in counseling sessions.

Information about the availability of a new contraceptive option was shared through posters, leaflets, health fairs and radio spots. In India, information was also disseminated through wall paintings, street theatre and puppet shows.

### Survey design

Study design was quasi-experimental with nonequivalent control (control and intervention sites had somewhat different characteristics at baseline). However, much of the analysis was focused on intervention sites at endline, because there was no Standard Days Method activity at baseline and in control areas. The quasi-experimental aspect of the study was necessary to examine changes in contraceptive prevalence and the method mix. The household-based survey focused on married women of reproductive age; a male survey (men married to women of reproductive age) was conducted in parallel. A household listing was used to randomly select households in intervention and control communities. All married women of reproductive age within the selected households were interviewed. Men were interviewed in 50% of the selected households (randomly selected, couples not matched).

In India and Peru, household-based surveys with women and men were undertaken before the start of intervention activities (baseline) and two years later (endline). In Rwanda we only conducted the survey at endline, and only in the intervention area, because contraceptive prevalence was low, and we could not obtain a sufficient sample size to show change in contraceptive prevalence over time, or between intervention and control. Due to a procedural error in the endline in India, sterilized women were erroneously excluded from the sample. To correct for this, we added a second endline in India, interviewing women only, nine months after the first. We present results from both endline surveys. The Georgetown University Institutional Review Board approved the survey protocol and instruments.

Sample size power calculations resulted in a desired sample size of 1710 women in India (570 per block), 1300 in Peru (650 each in control and intervention areas), and 600 women in Rwanda. The target sample size for men was half that of women.

The contraception module of the Demographic and Health Survey was adapted for the questionnaire, which included additional emphasis on Standard Days Method and was translated into Hindi, Spanish, and French. Respondents were asked about their background and contraceptive knowledge and use, including detailed questions on Standard Days Method. Descriptive and multivariate analyses compared intervention and control areas at baseline and endline.

### Service statistics

Service statistics were used to evaluate the effect of introducing Standard Days Method on the number of new family planning method users of all methods, including Standard Days Method. Mechanisms for collecting accurate service statistics on family planning use were improved in all participating facilities before data collection, and procedures for data verification were incorporated.

Data collection began in early 2005, three months before providers in intervention areas were updated on other family planning methods and trained to offer Standard Days Method, and continued for 18 additional months. Data included monthly figures of new family planning method users per method, regardless of whether they were new to family planning or switched from another method. For new Standard Days Method users (only), we collected information also on the method they switched from (if any). To look at data trends we aggregated the service statistics data into quarters.

## Results and discussion

We present our results as they relate to the policy questions raised by stakeholders that this study sought to address.

### What proportion of men and women in the community hear of Standard Days Method?

Table 2 presents the percentage of survey respondents who had ever heard of Standard Days Method at endline. In India we include both endlines – while participants in endline-1 were less representative of the population because female sterilization users were excluded, endline-2 occurred almost three years after the intervention began, and almost a year after direct Standard Days Method IEC activities ended, compared to endline-1, and the endlines in Peru and Rwanda, which took place about two years after the start of Standard Days Method introduction.

**Table 2:** Percentage of respondents who had heard of the Standard Days Method in the intervention areas at endline

	**India**			**Peru**		**Rwanda**	
	**Female**		**Male n = 692**				
	**Endline-1 n = 1165**	**Endline-2 n = 1202**		**Female n = 629**	**Male n = 524**	**Female n = 405**	**Male n = 211**
Spontaneous	15.3	4.4	6.9	35.5	23.9	65.4	38.4
Probed	43.9	36.8	31.5	27.7	15.0	25.2	30.8
Total	59.2	41.2	38.4	63.2	38.9	90.3	69.2

There is evidence of some contamination – a small number of respondents had heard of the method in the control areas at endline (2.7% of women in Peru; 7.0% and 4.0% in the two India endlines). Almost no respondent had ever heard of the method in intervention areas at baseline (0.4% in India; 0.2% in Peru).

The proportion of respondents who had heard of Standard Days Method in intervention areas in India and Peru was relatively high (59.2% in India endline-1 and 63.2% in Peru), considering that the method had never been offered or promoted in the intervention areas until our intervention. The proportion of women in Rwanda who had heard of Standard Days Method (90.3%) was significantly higher. The proportion of female respondents in India who had heard of the method in endline-2 was lower than in endline-1, probably because IEC and promotion efforts ended after endline-1. In all three study countries, fewer men than women had heard of the method.

Logistic regression analysis was conducted to determine if women with specific background characteristics were more likely to hear about the method than others (Table 3). The dependent variable was ‘heard of Standard Days Method’, coded 1 if yes (spontaneous or probed), 0 otherwise. In India and Peru younger women were less likely to be aware of Standard Days Method. Literate respondents were 1.5-3 times more likely to have heard of the method in all three countries. Finally, in India endline-2 and Rwanda, women who worked for money were more likely to have heard about the method.

**Table 3:** Odds ratio of logistic regression of hearing about the Standard Days Method in intervention areas at endline (women)

	**India**		**Peru**	**Rwanda**
	**Endline-1 n = 1154**	**Endline-2 n = 1199**	**n = 710**	**M = 405**
Age	0.947**	0.926**	0.952**	0.974
Number of children	1.028	1.167**	1.005	1.105
Literate	1.669**	2.159**	2.228**	3.260**
Catholic			1.348	1.949
Hindu	0.828	0.957		
Works for money	1.083	1.342**	1.227	3.755**
Wants to have another child	1.065	0.809	1.229	1.122
Constant	5.301**	1.745	2.761*	2.155
−2 log likelihood	1507.832	1370.946	882.811	221.467

* p < 0.05; ** *p* < 0.01.

### How many clients choose Standard Days Method compared to established methods?

Table 4 shows the percent of survey respondents in the intervention areas at endline, who had ever used Standard Days Method, and who were using it at the time of the survey. Our results show substantial uptake of Standard Days Method in all three countries. The lower figures in India endline-2 compared to India endline-1 reflect the exclusion of sterilized women from the sample in endline-1 (the proportion of users of other methods (including Standard Days Method) was larger). Very few respondents in the control areas had ever used the method (2.8% of women in India endline-1; 0.2% of women in Peru).

**Table 4:** Percentage using the Standard Days Method in intervention sites at endline

	**India**			**Peru**		**Rwanda**	
**Score**	**Female**		**Male n = 634**	**Female n = 629**	**Male n = 564**	**Female n = 405**	**Male n = 211**
	**endline-1 n = 1169**	**endline-2 n = 1202**					
Ever used Standard Days Method	6.1	3.9	7.9	5.2	5.6	5.1	3.3
Currently using Standard Days Method	5.0	1.2	4.7	3.8	4.1	0.5	2.4

**Figure 1:**
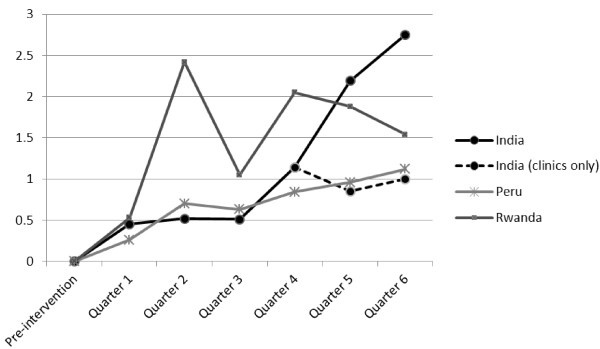
Average number per service delivery point of new Standard Days Method users by three-months periods (quarters).

We examined service statistics to more directly measure uptake of Standard Days Method. The average number of new Standard Days Method users per participating clinic is presented in Figure 1. Community health workers not associated with health facilities also were considered ‘facilities’. Each quarter is a three month period, following the beginning of the intervention, when providers were first trained in Standard Days Method and received refresher training on other established methods. We did not expect to see any Standard Days Method uptake before the intervention started, because providers had not yet been trained in the method (most had never heard of it), and because the method was not yet included in facility records. Therefore the number of new method users before the intervention is 0 everywhere.

The number of new Standard Days Method users in India continually grew, especially in quarter 5 and 6 when community (Anganwadi) workers and NGO animators were trained. The dotted line refers to the number of users reported by health facilities only; it shows that clinics continued to report new Standard Days Method users after community workers were trained, but the number of new clinic users leveled off. Peru exhibited a continual growth in the number of new Standard Days Method users. Rwanda presented a different picture, with spikes which may reflect changes in IEC strategy.

### Does introducing Standard Days Method result in increased overall family planning use in MOH facilities?

**Figure 2:**
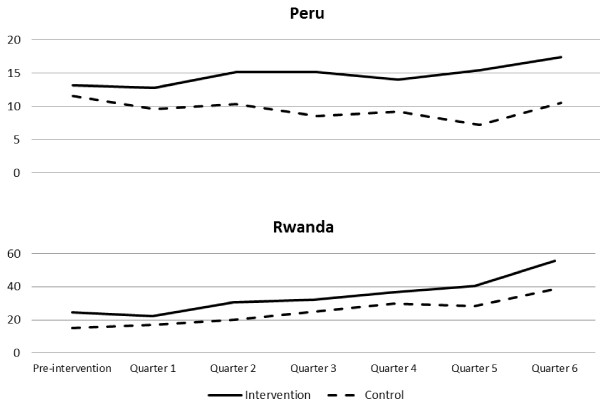
Average number per service delivery point of new family planning users by quarter, Peru and Rwanda.

We examine the effect of Standard Days Method introduction on overall contraceptive use. Figure 2 shows the number of new users per facility in Peru and Rwanda.

In Peru the number of new users decreased from baseline to quarter 6 in the control area but increased in the experimental area. This effect is not necessarily a result of the presence of the new method alone. Other aspects of the intervention or exogenous factors may have influenced the observed changes. In Rwanda the number of new users of all methods increased in both the control and intervention areas, consistent with the government commitment to increase overall contraceptive use.

**Figure 3:**
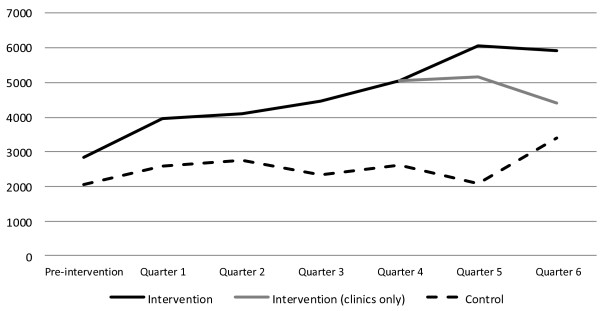
Number of new family planning users by quarter, India.

In India (Figure 3) new sterilization users were excluded from the figure because sterilizations are done periodically, in sterilization camps. Including them would skew the results. In addition in India, total numbers of users are shown instead of numbers per clinic, because community health workers were included (each individual community health worker was considered a ‘facility’). The higher number of new users in the pre-intervention quarter in the intervention areas reflects the much larger number of facilities in the two intervention blocks, compared to the control block. The number of new users increased in both the intervention and control areas, but more than doubled in the intervention areas, increasing by a smaller margin in the control block.

Returning to the survey we examine contraceptive prevalence, comparing the percent of respondents who used a modern family planning method^a^ in the baseline and endline surveys in the control and intervention areas. In India, there was a small increase in contraceptive prevalence from baseline to endline in the control area (not statistically significant). In the intervention area there was a statistically significant increase from 47.8% to 50.8%, suggesting an increase in contraceptive prevalence following Standard Days Method introduction. In Peru, there was a statistically significant increase in contraceptive prevalence in both the control and intervention areas. While we cannot assert that the increase is a result of the intervention, we can state that contraceptive prevalence did not decrease as a result of Standard Days Method introduction, a common concern voiced by policy makers.

### Are Standard Days Method users new to family planning, or do they shift from established methods?

Program managers are often concerned that introducing Standard Days Method will cause women to switch from already established, effective, methods. To address this concern, we determined whether new Standard Days Method users switched from another method, and if so, from which method.

In India, over 85% of new Standard Days Method users had never previously used a family planning method (not even a traditional method). Over 90% of new Standard Days Method users in Rwanda, and 57% in Peru, had not been using another method in the two months preceding their decision to use Standard Days Method. Table 5 shows the mean monthly number of new Standard Days Method users in quarter 6 who switched from another method, by method.

**Table 5:** Method that new Standard Days Method users switched from (Quarter 6)

**Method switched from**	**India**	**Peru**	**Rwanda**
Switched from DMPA	0.01	0.09	0.02
Switched from OC	0.17	0.39	0.02
Switched from Condoms	0.23	0.43	0
Switched from LAM	0	0.02	0.08
Switched from Billings Ovulation Method	0	0.03	0
Switched from Calendar Rhythm	0	0.02	0
Had never used a family planning method	2.68		
Had not used a family planning method in the two months prior to accepting Standard Days Method		1.32	1.98
Total	3.07	2.31	2.10

## Conclusions

Our findings provide information on the effect of introducing Standard Days Method into existing family planning services in India, Peru, and Rwanda, at both clinic and community levels. Service statistics show that in India and Peru Standard Days Method accounted for 12% and 13% of all new temporary method users in quarter 6; in Rwanda it accounted for 4% of new users. Examining the numbers of new users of all methods in the control and intervention areas in Peru suggests that the total number of family planning users did increase, perhaps as a result of Standard Days Method introduction. In India and Rwanda, however, the intervention had no effect (positive or negative) on the number of new family planning users at the clinic level, but results from the community survey in India show a possible positive effect of the intervention on contraceptive prevalence.

Successful introduction of a new method depends on raising awareness among potential users about the availability of a new option. A considerable percentage of women had heard of Standard Days Method in India and Peru. In Rwanda 90% of women had heard of the method, possibly because it had been available in the country for some time before the study, and the country is small. Multivariate analysis of Standard Days Method awareness suggests that older, less literate women were less likely to be aware of the method than their younger, better educated counterparts; fewer men had heard of the method than women. Future Standard Days Method introduction activities would benefit from new strategies to reach these segments of the population.

Results of this study have implications for larger-scale efforts to introduce Standard Days Method into family planning programs. A major impetus for the study was the need to address the questions policy makers raise when considering the introduction of the method into services. Data from these three diverse countries suggest that there is demand for Standard Days Method, and that this demand can increase over time as availability and awareness of the method increase. Perhaps most importantly, results demonstrate that Standard Days Method is most popular among women not currently using an effective method, thus providing programs a strategy to reach underserved couples. Standard Days Method can be a positive addition to the contraceptive mix, and has the potential of benefiting men and women in diverse settings and populations.

This study illustrates the critical role of evidence in moving an innovation to scale-up. While earlier clinical trials and pilot studies tested Standard Days Method efficacy and guided development of protocols and procedures for offering the method, this research provides evidence to address stakeholder concerns regarding the effect of Standard Days Method introduction on the broader system. Although Standard Days Method was introduced in a relatively small geographic area in each of the three countries, it was integrated into regular service delivery systems on a larger scale and with less technical assistance than previous studies. Evidence generated in this study not only addresses policy concerns, but also provides programmatic guidance.

## Endnotes

^a^Including Standard Days Method, sterilization, contraceptive injection, oral contraceptive, IUD, implant, condoms, and LAM.

## Competing interests

The author(s) declare that they have no competing interests.

## Authors’ contributions

RL was part of the team that conceived of the study, participated in its design and coordination, and helped draft the manuscript; IS was part of the team that conceived of the study, participated in its design, performed statistical analysis and helped draft the manuscript; PJ designed and coordinated study activities in India; MM designed and coordinated study activities in Rwanda; LS designed and coordinated study activities in Peru; FL participated in study design and performed statistical analysis. All authors approved of this version of the manuscript.
